# Post-translational modifications as a regulatory code for tau function in health and disease

**DOI:** 10.3389/frdem.2026.1834737

**Published:** 2026-06-03

**Authors:** Abigail J. Nordbeck, Bhirisha Sharma, Charles A. Garcia, Jui-Heng Tseng

**Affiliations:** 1 Arizona State University-Banner Neurodegenerative Disease Research Center at the Biodesign Institute, Tempe, AZ, United States; 2 School of Life Sciences, Arizona State University, Tempe, AZ, United States

**Keywords:** acetylation, Alzheimer’s disease, phosphorylation, post-translational modification, tau, ubiquitination

## Abstract

Tau is an intrinsically disordered microtubule-associated protein that performs diverse roles in neuronal physiology, including regulation of microtubule stability, intracellular transport, and synaptic signaling. These functions are dynamically regulated by an extensive array of post-translational modifications (PTMs) that collectively shape tau conformation, interactions, localization, and turnover. Under physiological conditions, PTMs act as a regulatory system that enables tau to transition between functional states in response to cellular cues. In neurodegenerative diseases collectively known as tauopathies, however, this finely balanced modification landscape becomes disrupted, leading to tau mislocalization, impaired clearance, and assembly into toxic oligomers and fibrillar aggregates. Although phosphorylation has historically dominated the tau field, growing evidence indicates that multiple PTMs, including acetylation, ubiquitination, truncation, oxidation, nitration, methylation, and glycosylation, cooperatively influence tau structure and pathogenic potential. Recent proteomic studies reveal that tau can harbor dozens of modifications simultaneously, highlighting the importance of understanding PTMs as an integrated regulatory network rather than independent events. Crosstalk between modifications can generate synergistic or antagonistic effects that influence tau aggregation, proteostasis, and propagation. In this review, we synthesize current knowledge of major tau PTMs and highlight emerging principles governing their interactions. We discuss how dysregulation of PTM networks contributes to tau state transitions during aging and neurodegeneration and consider how targeting PTM-regulating enzymes may provide therapeutic strategies for Alzheimer’s disease and related tauopathies.

## Introduction

Tau is an intrinsically disordered, microtubule-associated protein whose functional versatility arises from its conformational flexibility. In healthy neurons, tau dynamically associates with microtubules to regulate axonal stability, intracellular transport, and neuronal polarity (Weingarten et al., 1975; Cleveland et al., 1977; Wang and Mandelkow, 2016). While this microtubule-binding function has historically defined tau biology, it is now clear that tau operates beyond the axon as a multifunctional signaling and scaffolding protein (Mueller et al., 2021; Tseng and Cohen, 2024). Beyond microtubule binding, tau participates in a wide range of cellular processes, including synaptic signaling, nucleic acid protection, stress granule dynamics, and organelle organization (Ittner et al., 2010; Apicco et al., 2018; Tracy and Gan, 2018; Meier et al., 2015). These diverse functions require precise spatial and temporal control of tau interactions, allowing neurons to adapt to cellular cues, neuronal activity, and environmental stressors.

In the human brain, six tau isoforms are generated through alternative splicing of the *MAPT* gene at exons 2, 3, and 10 (Corsi et al., 2022). Splicing of exons 2 and 3 determines the number of N-terminal inserts (0 N, 1 N, or 2 N), whereas the inclusion or exclusion of exon 10 produces isoforms containing three (3R) or four (4R) microtubule-binding repeats. In the healthy adult brain, 3R and 4R tau are expressed at approximately equal levels, although the relative abundance of individual isoforms differs. Notably, 1N3R and 1N4R tau are enriched in adult brains, whereas 2N3R and 2N4R are the least prevalent (Buchholz and Zempel, 2024). In contrast, during early developmental stages, tau expression is restricted predominantly to the 0N3R isoform (Andreadis, 2005).

Post-translational modifications (PTMs) serve as a primary mechanism by which the functional and molecular properties of tau are regulated under physiological conditions (Wang and Mandelkow, 2016). Intrinsically Disordered Proteins (IDPs), like tau, are particularly susceptible to PTMs (Alquezar et al., 2020). Tau harbors a notably high density of modifiable residues and is subject to a broad spectrum of PTMs, including, but not limited to, phosphorylation, acetylation, ubiquitination, truncation, methylation, glycosylation, SUMOylation, nitration, and oxidation (Alquezar et al., 2020; Wesseling et al., 2020). Rather than acting as binary on–off switches, these modifications collectively shape tau conformation, binding affinity, subcellular localization, and turnover (Tseng and Cohen, 2024). In this way, PTMs enable tau to transition between functional properties that support neuronal homeostasis.

In tauopathies, including Alzheimer’s disease (AD), frontotemporal lobar degeneration (FTLD), chronic traumatic encephalopathy (CTE), progressive supranuclear palsy (PSP), corticobasal degeneration (CBD), and Pick’s disease (PiD), the normally tightly tuned landscape of tau PTMs becomes profoundly dysregulated (Arakhamia et al., 2020). Phosphorylation, along with other PTMs, are widely implicated in driving tau pathology across these disorders (Shoeibi et al., 2019; Kouri et al., 2011; Probst et al., 1996; Kumar et al., 2026). Despite this shared molecular feature, tauopathies are distinguished by their tau isoform composition and pathological signatures. For example, PSP and CBD are characterized by predominant 4R tau accumulation, whereas PiD is defined by 3R tau, suggesting that isoform-specific PTM patterns may contribute to selective vulnerability and aggregation behavior. Pathological tau is further characterized by aberrant modification patterns, mislocalization from axonal to somatodendritic compartments, impaired clearance, and self-assembly into toxic oligomers and fibrillar aggregates (Iqbal et al., 2010). Notably, in AD, the burden and spatial distribution of modified tau species correlate more closely with synaptic dysfunction and cognitive decline than amyloid pathology, underscoring tau as a central driver of neurodegeneration (Nelson et al., 2012).

Detection of protein PTMs relies on complementary methodological approaches. Mass spectrometry provides high-resolution, unbiased mapping and quantification of PTMs across tau isoforms (Huiwen Lei et al., 2026). In parallel, antibody-based techniques, including Western blotting, immunohistochemistry, and immunofluorescence, enable detection of site-specific modifications using well-characterized PTM-directed antibodies, particularly for phosphorylation and acetylation (Alhadidy and Kanaan, 2024).

Historically, tau phosphorylation has dominated the field, largely due to its abundance in neurofibrillary tangles (NFTs) and its impact on microtubule binding. However, growing evidence indicates that non-phosphorylation PTMs are equally critical in rewiring tau properties (Tseng and Cohen, 2024; Wesseling et al., 2020; Kumar et al., 2026). Acetylation can inhibit tau degradation and promote aggregation (Oh and Shin, 2025), ubiquitination regulates proteostatic routing (Jia and Fu, 2025), and proteolytic truncation generates tau fragments with enhanced seeding capacity and neurotoxicity (Chu et al., 2024). Oxidative and nitrative modifications further connect tau pathology to aging-related cellular stress, mitochondrial dysfunction, and redox imbalance (Perluigi et al., 2024). Tau is a classic neuronal IDP that lacks stable secondary and tertiary structure, and its electrostatic features support weak multivalent interactions and liquid–liquid phase separation (Alquezar et al., 2020; Huiwen Lei et al., 2026; Abasi et al., 2024). PTMs have been implicated in driving liquid–liquid phase separation (LLPS) with phosphorylated tau undergoing LLPS at an enhanced rate, potentially serving as an intermediate state between soluble tau and pathological aggregation (Wegmann et al., 2018). These findings support a model in which tau pathology emerges not from a single dominant modification, but from coordinated shifts in PTM networks.

**Figure 1:**
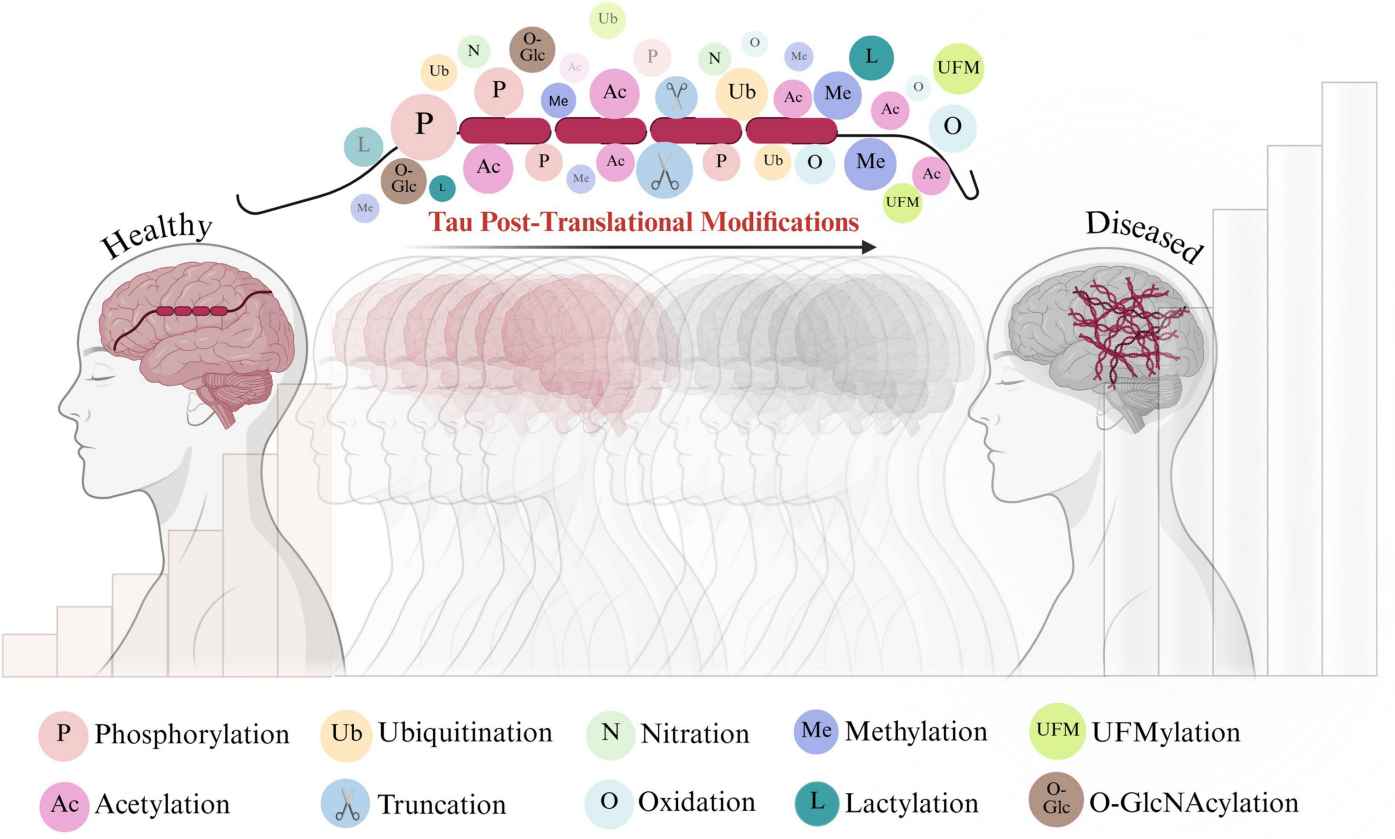
Conceptual model of the dynamic tau PTM network across the human lifespan. Schematic illustrating the dynamic and combinatorial landscape of tau post translational modifications (PTMs) during human aging. Multiple PTMs interact to regulate tau conformation, interactions, and functional configurations in a context-dependent manner. Rather than acting as binary switches, these modifications form an interconnected network that modulates tau behavior across physiological and pathological conditions. Dysregulation of this network may shift tau toward pathogenic conformations associated with tauopathies. PTM placement is conceptual and does not represent exact modification sites. Created with BioRender.com.

An emerging concept is that tau PTMs act combinatorially to reprogram tau function and biophysical properties across health and disease. Rather than acting as independent events, these modifications form an interconnected regulatory network that governs tau transitions (Figure 1). Crosstalk between modifications can determine whether tau remains soluble or aggregation-prone, whether it is efficiently cleared or persistently accumulates, and whether it adopts inert or prion-like conformations capable of propagation. Age-related changes in enzymatic activities, metabolic state, and proteostasis capacity may bias tau toward pathological PTM signatures, thereby lowering the threshold for disease onset and progression.

**Figure 2:**
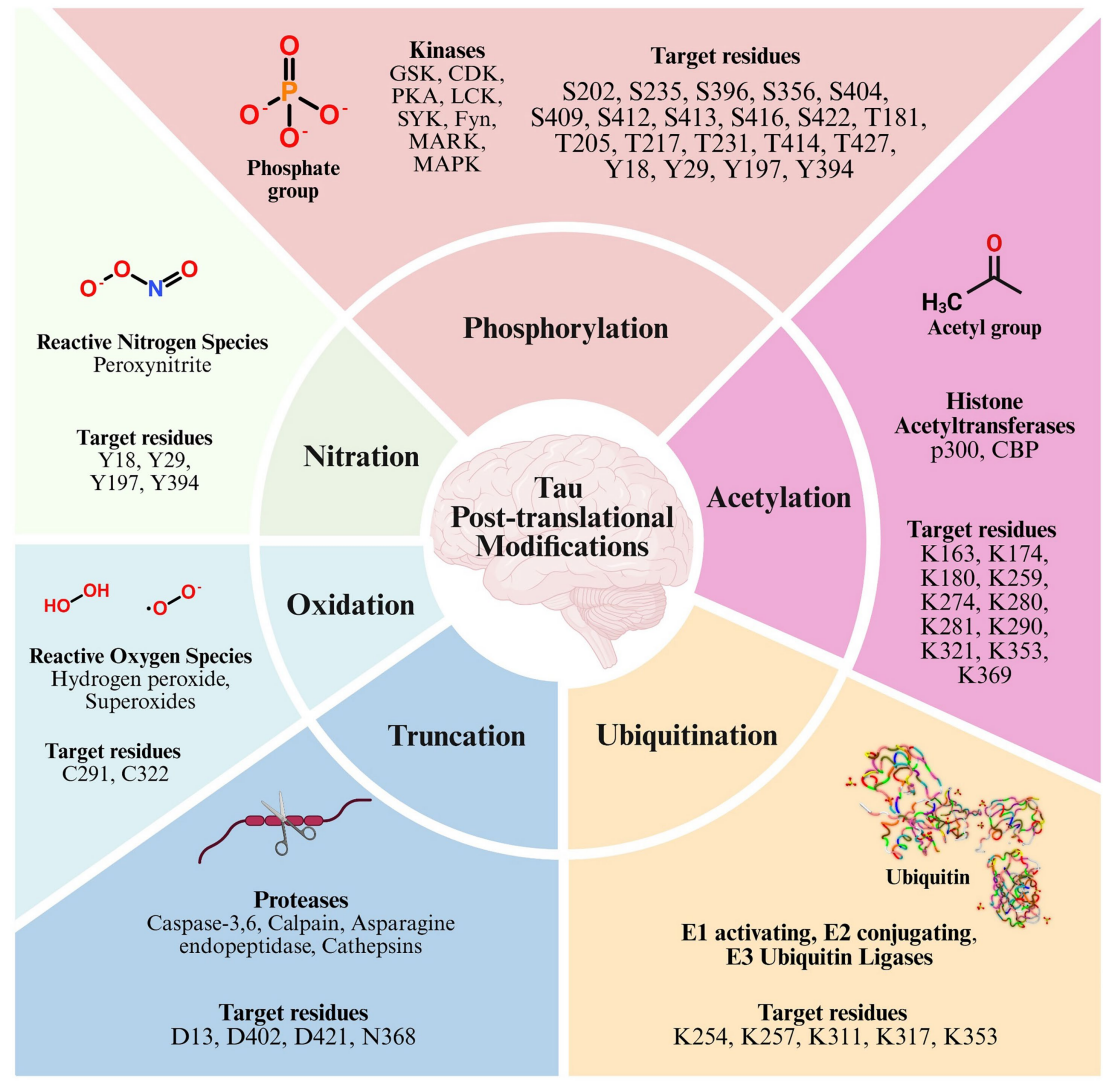
Major tau post-translational modifications, modifying enzymes, and representative target residues. Overview of major tau PTMs discussed in this review: phosphorylation, acetylation, ubiquitination, truncation, oxidation, and nitration. Modifying enzymes or reactive species are shown alongside representative target residues on tau, with chemical groups sourced from PubChem and the ubiquitin structure obtained from the Protein data Bank (PDB ID: 5O44). Only PTMs, enzymes, and sites discussed in this review are depicted. Created with BioRender.com. Adapted from a template by Wendy Jiang.

In this review, we discuss how tau PTMs rewire tau function, structure, and pathogenic potential across physiological and disease contexts. We summarize current knowledge on major tau PTMs, highlight emerging principles of PTM crosstalk and transitions, and discuss how dysregulation of these processes contributes to neurodegeneration. Major tau PTMs, their modifying enzymes, and representative modification sites are summarized in Figure 2, with a brief overview found in Table 1.

**Table 1:** Tau post-translational modifications with established biological and clinical relevance.

Residue	Modification	Relevance
S262	Phosphorylation	Reduces tau-microtubule binding
K280	Acetylation	
K281	Acetylation	
S356	Phosphorylation	
T181	Phosphorylation	Fluid biomarker for Alzheimer’s disease
T217	Phosphorylation	
T231	Phosphorylation	
S235	Phosphorylation	
S202	Phosphorylation	Marker for tau pathology
T205	Phosphorylation	
S396	Phosphorylation	
S404	Phosphorylation	
D421	Truncation	

Overview of selected tau PTMs with established biological and clinical relevance in AD. The table summarizes representative modification sites, PTM types, and their functional, clinical, and pathological relevance.

## Phosphorylation

Protein phosphorylation is the most extensively studied PTM, with the majority of work focused on canonical hydroxyl-containing residues serine, threonine, and tyrosine; however, phosphorylation chemistry extends beyond these classical sites. Among the 20 standard amino acids, nine can undergo phosphorylation through four classes of phosphate linkages collectively described as SONA (S-, O-, N-, and A-linked) (Zhao et al., 2025; Thibault Houles and Roux, 2024; Ardito et al., 2017). Through reversible addition of a phosphate group, phosphorylation rapidly remodels protein charge, conformation, interaction capacity, and subcellular localization (Alquezar et al., 2020; Zhao et al., 2025; Thibault Houles and Roux, 2024; Ardito et al., 2017; Jin and Grater, 2021). In addition to serine, threonine, and tyrosine, phosphorylation has been documented on noncanonical residues including arginine, lysine, aspartate, glutamate, histidine, and cysteine (Thibault Houles and Roux, 2024; Shanshan Wang et al., 2022; Hardman Gemma et al., 2019). Although diverse chemistries contribute to these reactions, O-phosphorylation on serine, threonine, and tyrosine remains the dominant form in eukaryotic signaling networks (Shanshan Wang et al., 2022; Anton Kalyuzhnyy et al., 2022). Phosphorylation is catalyzed by kinases and reversed by phosphatases, with serine sites representing the majority of the events, whereas threonine and tyrosine account for approximately 3–4% and less than 1%, respectively (Alquezar et al., 2020). Consistent with its central regulatory role, the human genome encodes more than 500 kinases that influence processes ranging from growth and differentiation to migration and apoptosis (Axtman, 2021; Berginski et al., 2020).

Within neurons, tau operates within a highly organized cytoskeletal network composed of microtubules, intermediate filaments, and actin filaments that collectively maintain neuronal morphology and polarity (Tseng and Cohen, 2024; Leterrier, 2021). First described in the 1980s (Grundke-Iqbal et al., 1986), tau phosphorylation is now recognized at more than 85 potential sites in the longest human tau isoform (2N4R), including five tyrosine residues, with the remainder distributed across serine and threonine residues (Alquezar et al., 2020). Experimental validation supports approximately 45 phosphorylation sites, which cluster predominantly within the proline-rich region and near the microtubule-binding regions (MTBR) (Tang et al., 2025; Wegmann et al., 2021). Physiological phosphorylation contributes to microtubule dynamics and axonal transport, whereas hyperphosphorylation reduces microtubule binding and promotes tau aggregation and NFT formation (Martin et al., 2013). Pathological phospho-epitopes such as S202/T205 (AT8) and S396/S404 (PHF-1) remain widely used markers of tau pathology in tauopathy brain (Wesseling et al., 2020). Multiple kinases contribute to these phosphorylation patterns, including GSK3β, CDK5, PKA, and CK1, while PP2A is widely considered the dominant tau phosphatase in the brain (Parra Bravo et al., 2024b).

Early pathological studies revealed that tau phosphorylation in AD follows a sequential and spatially organized trajectory rather than occurring randomly (Wang et al., 2002; Ikura et al., 1998). Phosphorylation at S202 catalyzed by p42 MAP kinase appears early within dystrophic neurites, later extending to neuronal soma, and is followed by phosphorylation at S396 within neurites and NFTs (Su et al., 1994). This progression suggests that early phosphorylation events may prime tau for substantial structural remodeling and cytoskeletal destabilization (Su et al., 1994). Building on these observations, more recent work has begun to define individual phosphorylation sites as regulatory switches, priming events, or potential therapeutic targets.

Several examples illustrate how site-specific phosphorylation integrates disease stage and cellular context. Phosphorylation at S356, mediated by NUAK1 kinase, correlates with Braak staging, accumulates within NFTs, and co-localizes with synaptic compartments, supporting a link to synaptic dysfunction (Taylor et al., 2024). Pharmacological inhibition of NUAK1 using NUAK kinase inhibitor WZ4003 reduces S356 phosphorylation in *ex vivo* human brain slices, and alters total tau levels in murine models, highlighting the feasibility of site-directed intervention (Taylor et al., 2024). In tauopathy models, blockade of T231 phosphorylation prevents propagation of downstream hyperphosphorylation, reduces aggregation and neurotoxicity, and improves behavioral outcomes, consistent with a gatekeeper role for T231 in maintaining a microtubule-binding-competent tau conformation (Stefanoska et al., 2022). Complementary phosphorylation-mimic studies in HT22 cells further suggest that phosphorylation can impair mitochondrial bioenergetics, lower ATP production, increase oxidative stress, and disrupt axonal mitochondrial transport in a site-dependent manner (Isei et al., 2024).

Regional and activity-dependent phosphorylation programs further underscore the context sensitivity of tau regulation. In the entorhinal cortex, a region vulnerable early in AD, cAMP-dependent protein kinase-driven phosphorylation at T231 and S235 correlates with pre-tangle conformational changes and cognitive decline, potentially contributing to early circuit vulnerability and spread of pathology into hippocampal regions (Reyes-Pablo et al., 2025). Phosphorylation can also intersect with proteostasis pathways. MARK2 phosphorylation of caspase-cleaved tauC3 at S416 weakens interaction with the E3 ligase CHIP, inhibiting degradation and promoting pathological persistence in AD models (Nadel et al., 2024). Neuronal hyperexcitation induces a distinct cluster of C-terminal phosphorylation events, including S409, S412, S413, T414, S416, S422, and T427, identified by unbiased mass spectrometry in hyperactive hippocampal neurons and attributed to Plk2 kinase (Schneeweis et al., 2025). These activity-dependent modifications promote dendritic re-localization of tau, alter postsynaptic density structure, and contribute to homeostatic synaptic scaling (Schneeweis et al., 2025).

Tau is also phosphorylated on tyrosine residues by multiple tyrosine kinases, including SRC family members LCK, SYK, Fyn, and ABL family members ABL1 and ARG (Alquezar et al., 2020; Stevenson et al., 2024). Phosphorylation at Y18, Y197, and Y394 has been associated with enhanced aggregation and AD-related pathology (Alquezar et al., 2020; Ait-Bouziad et al., 2020). In P301S tau transgenic mice, a model of tauopathy marked by age-dependent tau hyperphosphorylation and aggregation, TYK2-mediated phosphorylation at Y29 exacerbates pathological tau accumulation, whereas TYK2 knockdown reduces pathogenic tau and attenuates gliosis and disease progression (Kim et al., 2024). Y18 phosphorylation mediated by Fyn kinase can prevent exposure of the phosphatase-activating domain, thereby blocking activation of the PP1-GSK3 signaling cascade and protecting against tau-induced inhibition of anterograde fast axonal transport (Lee et al., 2004; Kanaan et al., 2012). These findings illustrate that tyrosine phosphorylation can modulate signaling and transport phenotypes in addition to influencing aggregation.

Recent studies have begun to map phosphorylation-dependent interaction networks in human disease. Affinity purification mass spectrometry in advanced AD brain identified an interactome associated with tau phosphorylation at T217, including multiple subunits of the CTLH E3 ubiquitin ligase complex, such as WDR26, ARMC8α/*β*, GID8, RANBP9, and MAEA (Kavanagh et al., 2025). These interactions suggest that T217 phosphorylation may redirect tau toward distinct degradation-related pathways. Phosphorylation at T217, along with T181, T231, and S235, is detected in tau NFTs, and these species have emerged as key biomarkers for AD, each reflecting distinct aspects of disease biology, including amyloid burden, early or incipient stages, and disease progression, with pT217 showing particularly strong association with disease severity (Lai et al., 2024; Ashton et al., 2021; Lantero-Rodriguez et al., 2021; Lleó et al., 2021). Notably, phosphorylation at T217 is not exclusively pathological. Multicenter clinical data show that plasma pT217 levels are elevated in healthy newborns and decline rapidly during early development, indicating that this modification may serve physiological roles in early life while becoming dysregulated in disease contexts (Gonzalez-Ortiz et al., 2025).

Despite extensive mapping of tau phosphorylation, the functional significance of many sites remains unresolved. Residues including S113, T153, S185, T175, S191, S237, and S289 are detected in pathological tau yet lack clear mechanistic interpretations (Wesseling et al., 2020; Kimura et al., 2024). Phosphorylation at S289 and S293 is particularly intriguing because both sites lie in the MTBR, where subtle charge changes could influence microtubule affinity or bias tau toward seeding-competent conformations (Man et al., 2023). However, direct functional studies remain limited, highlighting the need to move beyond site cataloging toward integrative mechanistic dissection in physiologically relevant systems.

## Acetylation

Acetylation is a reversible post-translational modification in which an acetyl group is transferred from acetyl-CoA to the *ε*-amino group of lysine residues, a reaction catalyzed primarily by lysine acetyltransferases (HATs), including p300, CBP, and TIP60 (Ali et al., 2018; Dancy and Cole, 2015). In chromatin, acetylation neutralizes the positive charge of lysine to relax chromatin and promote transcriptional activation, whereas histone deacetylases (HDACs) remove acetyl groups to condense chromatin and repress transcription. Beyond chromatin, acetylation remodels protein charge and can alter conformation, stability, protein–protein interactions, and subcellular localization (Cohen et al., 2011; Trzeciakiewicz et al., 2017). Acetyltransferases and HDACs together dynamically regulate acetylation across nuclear and cytoplasmic substrates, and lysine acetylation is observed in diverse protein classes, including transcription factors, metabolic enzymes, cytoskeletal proteins, and chaperones, underscoring its broad roles in cellular physiology (Kabir et al., 2022; Narita et al., 2019).

In the tau field, acetylation has emerged as a major regulatory modification that shapes tau function, stability, and pathogenicity in AD and related tauopathies (Tseng and Cohen, 2024; Alquezar et al., 2020; Cohen et al., 2011; Irwin et al., 2012; Irwin et al., 2013; Min et al., 2010; Cook et al., 2014). There are 44 lysine residues on tau that are susceptible to acetylation (Oh and Shin, 2025). In contrast to phosphorylation, acetylation prominently targets lysine residues within the MTBR and can uncouple tau from its canonical cytoskeletal functions while promoting pathological gain-of-function (Trzeciakiewicz et al., 2017; Irwin et al., 2012). Early studies demonstrated that acetylation at K280 inhibits tau microtubule binding and stabilization while enhancing fibrillization and aggregation (Trzeciakiewicz et al., 2017; Cook et al., 2014). *In vitro*, K280 acetylation impairs microtubule assembly and increases insoluble tau formation, and immunohistochemical analyses detect acetylated tau enrichment within neurofibrillary tangles, neuritic plaques, and related inclusions across AD, CBD, and PSP (Cohen et al., 2011). Together, these findings position tau acetylation as an early disease-relevant event that biases tau toward an aggregation-prone state.

Subsequent work has expanded this framework to additional lysine residues and clarified mechanisms linking acetylation to proteostasis failure and intercellular spread. P300/CBP-mediated acetylation at K174, K274, K280, and K281 has been associated with reduced microtubule binding, impaired clearance through proteasomal and chaperone-mediated autophagy pathways, and enhanced tau propagation across synapses (Oh and Shin, 2025; Wang et al., 2020). Acetylation at K174 and K281 has been reported to suppress mitochondrial fusion proteins, including Mfn1, Mfn2, and OPA1, promoting excessive fission, mitochondrial fragmentation, and bioenergetic decline in cellular and animal models, with downstream consequences for synaptic integrity and cognition (Zhang et al., 2024). Computational and *in vitro* studies further suggest that acetylation at K274 induces structural rearrangements that reduce microtubule affinity and favor aggregation-prone oligomers, increasing seeding potential while intersecting with tau clearance pathways through altered chaperone engagement (Zou et al., 2024). Collectively, these findings support a model in which acetylation rewires tau behavior through coordinated effects on microtubule binding, conformational state, organelle function, and proteostasis.

Emerging evidence highlights isoform- and site-specific effects of tau acetylation in regulating aggregation propensity. Biochemical analyses using acetylation-mimic (K to Q substitutions) indicate that K280Q in 4R tau accelerates aggregation compared with wild-type tau, whereas acetylation at K298 markedly delays fibril formation (Chakraborty et al., 2023). In contrast, studies in 0N3R tau revealed widespread acetylation across multiple lysine residues that promoted fibrillization, although precise site-specific quantification was limited due to overlapping NMR spectral signals (Chakraborty et al., 2023). These observations suggest that acetylation can exert divergent effects on tau aggregation depending on the modified residue and tau isoform, with certain sites destabilizing microtubule interactions and favoring aggregation-prone conformations.

Acetyltransferase activity has also been linked to tau secretion and propagation. Hyperactivation of p300/CBP impairs the autophagy-lysosomal pathway in a tau transgenic mouse model, leading to accumulation of autophagic markers, increased tau acetylation, and enhanced extracellular tau release (Chen et al., 2020). In cultured neurons, p300/CBP overactivation blocks autophagic flux and promotes fibril-induced tau spreading, whereas genetic or pharmacological inhibition of p300/CBP restores autophagic function, reduces intracellular tau accumulation, and attenuates tau propagation *in vitro* and *in vivo* (Chen et al., 2020). Notably, acetylation within the tau fibril core appears to exert limited or context-dependent effects on pathological seeding. In human P301L tau cellular seeding models, mutation of K311, K353, K369, K370, and K375 to alanine induced spontaneous amorphous aggregation that templated selectively to P301L tau (Smith et al., 2025).

Despite growing recognition that tau acetylation neutralizes lysine charge, disrupts microtubule binding, and promotes aggregation, many acetylation events remain insufficiently characterized with respect to their precise biological roles. For example, acetylation at K311 within the aggregation-prone VQIVYK motif has been implicated in modulating tau’s transition toward seed-competent conformations (Li et al., 2023), yet the mechanistic basis for this effect and its interplay with competing ubiquitination at the same residue remain poorly defined. Continued work will be required to define how site-specific acetylation integrates with broader PTM networks to shape tau transitions during aging and neurodegeneration.

## Ubiquitination

The ubiquitin-proteasome system (UPS) is the primary degradation mechanism of regulatory and damaged proteins that have been tagged with ubiquitin (Schmidt and Finley, 2014). Ubiquitination is a highly versatile post-translational modification executed through an enzymatic cascade involving ubiquitin activation (E1), conjugation (E2), and substrate-specific ligation by E3 ligases (Zhao et al., 2022; Tsai et al., 2024; Ikeda and Dikic, 2008; Yu et al., 2025). The human genome encodes eight distinct E1 enzymes, two of which initiate conjugation, around 40 E2 enzymes, and over 600 E3 ligases (Stratton et al., 2025; Sheng et al., 2024; George et al., 2018). E3 ligases contain one of three different catalytic domains, recognize different substrates, and catalyze different ubiquitin linkages, all of which determine the specificity and increase the diversity of ubiquitination (George et al., 2018). Ubiquitin signals are further regulated by deubiquitinating enzymes, which remove or remodel ubiquitin chains to shape downstream outcomes (Liu et al., 2024).

Ubiquitination plays an important role in cellular homeostasis by regulating cellular responses to exogenous stressors, such as hypoxia, heat shock, and endogenous stressors, including oxidative stress, endoplasmic reticulum (ER) stress, and DNA damage (Sheng et al., 2024). However, exogenous stressors may trigger endogenous stressors, which can lead to more intercellular responses, creating a complicated cascade for ubiquitin regulation. At the molecular level, monoubiquitination involves the formation of an isopeptide bond between the C-terminal carboxyl group of ubiquitin and the *ε*-amino group of a substrate lysine. Beyond single ubiquitin addition, ubiquitin itself can be ubiquitinated through any of its seven internal lysines (K6, K11, K27, K29, K33, K48, or K63) or via the N-terminal methionine (M1), greatly expanding the coding capacity of this modification (Damgaard, 2021). Polyubiquitin chains may be homotypic or mixed, depending on linkage composition (Musaus et al., 2020), and can form branched architectures in which individual ubiquitin molecules are modified at multiple positions, generating higher-order topologies with distinct signaling properties (Swatek and Komander, 2016; Komander and Rape, 2012). Linkage type and chain architecture, therefore, function as molecular codes that direct diverse biological outcomes (Ikeda and Dikic, 2008).

Tau is enriched in lysine residues, and in the 2N4R tau isoform, 17 of 44 lysines have been identified as ubiquitination sites, many of which overlap with acetylation and methylation (Morris et al., 2015; Munari et al., 2020). Importantly, ubiquitin signals on tau encode context-dependent biological outputs determined by residue location, linkage type, and chain topology. In tauopathy brains, ubiquitinated tau species are readily detected within pathological aggregates. Aggregated tau isolated from AD and related tauopathies exhibits monoubiquitination at K254, K257, K311, and K317, as well as polyubiquitination at K254, K311, and K353 (Morishima-Kawashima et al., 1993; Cripps et al., 2006). These sites cluster within or near the MTBR, supporting the concept that ubiquitination can directly influence tau’s canonical cytoskeletal functions. Indeed, monoubiquitination within the MTBR reduces tau’s affinity for microtubules, providing a plausible mechanism by which early ubiquitin signaling destabilizes axonal structure (Cripps et al., 2006).

Detailed analysis of paired helical filaments (PHFs)-associated tau further suggests that ubiquitination occurs early during aggregation formation (Cripps et al., 2006). Cripps et al. (2006) reported that soluble PHF-associated tau is polyubiquitinated at K254, K311, and K353, and decorated with multiple ubiquitin linkage types dominated by K48-linked chains with additional K6 and K11 linkages. These findings introduce a central paradox in tau biology. K48-linked ubiquitin chains are widely regarded as canonical signals for 26S proteasome-mediated degradation (Yu et al., 2025), yet ubiquitinated tau accumulates rather than being efficiently cleared. This accumulation likely reflects impairment of proteostatic systems, including proteasomal and lysosomal pathways, during disease progression, rather than active evasion of degradation by polyubiquitinated tau.

Additional linkage types present on pathological tau may further shape clearance and signaling outcomes. K6-linked ubiquitination has been associated with enhanced susceptibility to oxidative stress, reduced degradation efficiency in certain contexts, and may interfere with proteasomal processing, although its direct role in tau pathology remains unclear (Shang et al., 2005). K11-linked ubiquitination is often discussed in the context of proteolytic targeting (Matsumoto et al., 2010; Qin et al., 2014), yet the proteasome displays relatively weak affinity for purely homotypic K11 chains, suggesting that mixed architectures or additional regulatory inputs may be required to drive efficient degradation. One study found unconventional linkages, such as K11, may accumulate with ER stress and may target proteins for degradation (Xu et al., 2009). However, unconventional ubiquitin linkages are not well understood, and their role in the UPS and cellular homeostasis requires further investigation. Together, these observations support a model in which tau ubiquitination operates as a multifaceted signal integrating degradation, trafficking, and stress responses rather than a simple binary clearance tag.

Beyond degradation-associated linkages, K63-linked ubiquitination represents a major degradation-independent signal with broad roles in cell signaling and proteostasis (Musaus et al., 2020). K63 chains regulate kinase activation pathways (Chen and Sun, 2009), including NF-κB signaling (Wang et al., 2012), and influence neuronal functions such as synaptic remodeling and memory formation (Dresselhaus and Meffert, 2019). They also participate in DNA repair (Lee et al., 2017), intracellular trafficking (Erpapazoglou et al., 2014), endocytosis (Tanno and Komada, 2013), autophagy (Shaid et al., 2013), and mitophagy (Cunningham et al., 2015), pathways frequently disrupted in tauopathies. Consistent with these roles, K63 ubiquitin immunoreactivity has been observed in AD brain within perisomatic bodies, neuropil, and neurofibrillary tangles (Paine et al., 2009). Puangmalai et al. (2022) further demonstrated that K63-modified soluble tau oligomers accumulate in the AD brain and exhibit enhanced seeding activity and propagation in primary neurons. While these findings link K63 ubiquitination to tau strain-like behavior and spread, the mechanistic basis by which K63 modification interfaces with stress signaling and cell death pathways remains incompletely understood.

Several linkage types remain comparatively understudied in tau biology. K27-linked ubiquitination has been implicated in proteasomal regulation (Birsa et al., 2014) and DNA damage responses, yet technical challenges in mapping K27-specific interactions have limited mechanistic insight (Gatti et al., 2015). K29-linked ubiquitination has been associated with the regulation of mRNA stability (Zhou et al., 2013), raising the possibility that K29-modified tau could influence local translation and synaptic function relevant to memory impairment in AD (Gupta-Agarwal et al., 2014; Schaefer et al., 2009; Gupta-Agarwal et al., 2012). K33-linked ubiquitination is most often associated with intracellular trafficking (Yuan et al., 2014), although select studies suggest context-dependent roles in proteostasis (Kim et al., 2013; Michel et al., 2015). Direct evidence for these functions in tau biology remains limited.

Finally, ubiquitin signaling becomes markedly more complex when mixed and branched chain architectures are considered. While many homotypic chains are relatively well characterized, the structure and outcomes of mixed or branched configurations- including mixed M1/K63, and branched K48/K63, or K11-containing chains- are only beginning to emerge (French et al., 2021; Meyer and Rape, 2014). Whether tau is modified by these higher-order architectures in neurons, and how such topologies influence tau localization, turnover, and state transitions, remain important open questions. Overall, a substantial gap persists between descriptive mapping of ubiquitin marks on tau and a mechanistic understanding of how specific linkage types reshape tau function during aging and neurodegeneration.

## Truncation/cleavage

Protein truncation, often referred to as proteolytic cleavage, is a common PTM in which proteases hydrolyze peptide bonds to generate shorter fragments with altered conformation, stability, localization, or function. Multiple protease families contribute to this process, including caspases, calpains, asparagine endopeptidase (*δ*-secretase/AEP), and cathepsins (Quinn et al., 2018). Under physiological conditions, tightly regulated cleavage supports cellular homeostasis by activating signaling molecules, exemplified by caspase-mediated cytokine maturation (Zhang et al., 2022), removing inhibitory or regulatory domains (Nguyen et al., 2021), generating bioactive peptides, and facilitating protein turnover (Park et al., 2020). In contrast, dysregulated proteolysis can be pathogenic, producing fragments that evade clearance, acquire aggregation propensity, or propagate pathology across tissues, with implications spanning neurodegeneration to cancer (Frankowska et al., 2022; Boyarko and Hook, 2021; Vizovisek et al., 2021). Within tau biology, cellular and murine studies indicate that N- or C- terminally truncated tau species disrupt mitochondrial bioenergetics, impair axonal transport, and induce neuronal injury phenotypes consistent with early neurodegenerative changes in AD (Olesen and Quintanilla, 2023; Opland et al., 2023).

Tauopathies provide a clear illustration of how proteolytic processing can shift from regulated control to a disease-amplifying mechanism (Yang et al., 2024). Tau can be cleaved by multiple proteases, including caspase-3 at D421, caspase-6 at D402 and D13, *δ*-secretase at sites such as N368, calpains, and additional enzymes, generating fragments that are often aggregation-prone, resistant to degradation, and capable of templating pathology in full-length tau. δ-secretase-mediated cleavage has been implicated in initiating tau aggregation within the locus coeruleus and promoting prion-like spread to anatomically connected brain regions in AD (Olesen and Quintanilla, 2023; Kang et al., 2020). Caspase-6-derived fragments accumulate disproportionately in AD (3R + 4R tauopathy) and Pick’s disease (3R tauopathy) compared with other 4R tauopathies and can be detected in neurons lacking overt hyperphosphorylated tau aggregates, suggesting that truncation may represent an early PTM in AD, which might be independent of tau phosphorylation (Theofilas et al., 2022). Caspase-3-cleaved tau at D421 generates truncated species that are particularly insoluble and neurotoxic. Antibody-based targeting of these fragments, including monoclonal antibody 5G2, restores neuronal function, reduces microglial activation, and promotes lysosomal degradation in preclinical models (Martín-Ávila et al., 2025).

Beyond individual cleavage epitopes, systematic analysis suggests that truncation broadly sensitizes tau toward pathogenic transitions. Deletion of the N-terminal 150 or 230 amino acids, or the C-terminal 50 amino acids, enhances site-specific phosphorylation, aggregation, binding to AD-derived oligomeric tau, and seeding activity *in vitro* and in cellular models, with Tau151–391 exhibiting particularly strong pathological potency (Gu et al., 2020). Recent work further links caspase-mediated cleavage to synaptic dysfunction independent of late-stage tangle burden. Opland et al. (2023) reported that proteasome impairment drives activity-dependent accumulation of caspase-3-cleaved tau at residue D421within the post-synaptic density, where this truncated species disrupts neuronal firing and impairs network burst initiation, consistent with reduced excitatory drive.

Collectively, these findings support a model in which proteolytic cleavage fulfills physiological roles when tightly regulated but becomes pathogenic when tau-cleaving protease activity is dysregulated. In disease contexts, truncation generates toxic, aggregation-prone tau fragments that resist clearance, amplify seeding and spread, and contribute to synaptic and network dysfunction, positioning tau truncation as a mechanistically grounded therapeutic target in AD and related tauopathies.

## Oxidation/nitration

Oxidative stress reflects an age-associated imbalance in oxidation–reduction reactions that leads to the accumulation of reactive oxygen species (ROS) and reactive nitrogen species (RNS) (Commoner et al., 1954; Zorov et al., 2014; Phaniendra et al., 2015). These reactive intermediates include free radicals that harbor unpaired electrons and display a broad range of chemical reactivities and biological consequences (Commoner et al., 1954). In the nervous system, ROS and RNS arise from both endogenous sources, including mitochondrial metabolism and immune-related processes (Zorov et al., 2014), and exogenous factors such as environmental exposures (Phaniendra et al., 2015). At physiological levels, redox species participate in signaling and homeostatic regulation (Manful et al., 2025; Valko et al., 2007); however, when production exceeds antioxidant buffering capacity (Jakubek et al., 2024), oxidative and nitrosative stress can destabilize protein structure and cellular function.

Tauopathies are characterized by a reciprocal relationship between oxidative stress and tau dysfunction. Elevated ROS has been linked to tau hyperphosphorylation, reduced microtubule binding, and cytoskeletal destabilization, changes that promote tau mislocalization and aggregation into PHF and NFTs (Gu and Scarmeas, 2011; Delacourte, 2005; Chandran and Binninger, 2023). In turn, tau aggregation and disruption of microtubule-based transport impair mitochondrial function and proteostasis, further increasing ROS production (Gu and Scarmeas, 2011; Delacourte, 2005; Chandran and Binninger, 2023). This feedforward coupling provides a plausible mechanism by which age-related or transient redox imbalance can become self-sustaining during disease progression (Gu and Scarmeas, 2011; Delacourte, 2005; Chandran and Binninger, 2023).

At the molecular level, tau is susceptible to redox-linked PTMs, most prominently oxidation and nitration, which can directly reshape tau conformation and assembly behavior. Oxidation preferentially targets sulfur-containing residues such as cysteine and methionine, whose side chains readily react with reactive intermediates (Kim et al., 2014; Fra et al., 2017). The 2N4R tau isoform contains five methionine residues, and methionine oxidation has been observed under oxidative stress conditions (Beckman, 1996; Alvarez and Radi, 2003). However, the site specificity and functional consequences of methionine oxidation remain poorly defined. Strong oxidants such as peroxynitrite induce methionine oxidation alongside additional modifications, including tyrosine nitration and S-nitrosylation (Vana et al., 2011), complicating attribution of specific phenotypes to individual oxidative events. Defining which methionines are modified *in vivo* and how distinct oxidant species bias tau toward specific conformational configurations remains an important unresolved question.

Tau contains two cysteine residues, C291 and C322, that are particularly sensitive to oxidation (Morris et al., 2015). Cysteine oxidation can alter intramolecular and intermolecular disulfide chemistry, thereby regulating tau self-assembly. *In vitro* studies demonstrate that oxidation at C322 promotes tau aggregation into PHF-like structures (Schweers et al., 1995), whereas maintaining C322 in a reduced state, mutating cysteine residues, or favoring intramolecular disulfide bonding suppresses tau aggregation (Schweers et al., 1995; Bhattacharya et al., 2001). Beyond aggregation, cysteine redox state may intersect with tau turnover pathways. Recent evidence suggests that oxidation at C291 and C322 facilitates tau internalization through an endosomal mitophagy-associated route (Caballero et al., 2018), raising the possibility that cysteine oxidation can influence both tau accumulation and intercellular handling.

Nitration represents a parallel redox-linked modification that primarily targets tyrosine residues and is often associated with peroxynitrite-driven chemistry (Reynolds et al., 2007). Tyrosine nitration alters hydrogen binding, hydrophobicity, and electrostatic properties, with potential consequences for protein interactions and assembly. Early studies showed efficient peroxynitrite-mediated nitration of tau (Reynolds et al., 2005a) and reported inhibitory effects on tau filament formation (Reynolds et al., 2005b); however, subsequent work revealed that peroxynitrite induces multiple concurrent oxidative modifications, complicating interpretation of the specific contribution of tyrosine nitration alone (Vana et al., 2011).

Five tyrosine residues are present in the 2N4R tau isoform, with nitration reported at Y18, Y29, Y197, and Y394 *in vitro*. In human tissue, Y197 nitration has been detected in neurologically healthy brain (Reyes et al., 2011), whereas nitration at Y18, Y29, and Y394 is enriched in AD (Reyes et al., 2012). Notably, nitration at Y18 has been linked to astrocyte activation and early amyloid-associated changes (Reyes et al., 2008), suggesting that tau nitration may participate in early neuroinflammatory or stress-responsive pathways. These observations support the view that tau nitration occurs in both physiological and pathological contexts, with outcomes that depend on tau state, cellular compartment, and the broader redox environment.

Overall, oxidation and nitration intersect with tau biology at multiple levels, influencing aggregation propensity, microtubule interactions, and proteostasis (Schweers et al., 1995; Bhattacharya et al., 2001; Reynolds et al., 2005b). However, major gaps remain in defining residue-specific logic, temporal order, and crosstalk with other PTMs such as phosphorylation, acetylation, and ubiquitination. Rather than acting as isolated insults, redox modifications are likely to bias tau toward permissive or pathological modification landscapes by altering kinase activity, chaperone engagement, and clearance pathways (Gu and Scarmeas, 2011; Delacourte, 2005; Chandran and Binninger, 2023). Clarifying how oxidative stress reshapes tau’s PTM network will be essential for understanding how aging-related redox imbalance transitions tau from regulated function to neurodegenerative pathology.

## Other PTMs

### Methylation

Protein methylation represents a relatively subtle chemical modification compared with many other PTMs, as it does not introduce large chemical groups or substantial charge changes. Instead, methyltransferases transfer a methyl group from S-adenosylmethionine to lysine and arginine residues, producing S-adenosylhomocysteine as a byproduct (Di Blasi et al., 2021). Lysine residues can undergo mono-, di-, or tri- methylation, whereas arginine residues can form mono-methylated, symmetric di-methylated, or asymmetric di-methylated species (Di Blasi et al., 2021; Rowe et al., 2019). These reactions are catalyzed by lysine methyltransferases and protein arginine methyltransferases (Rowe et al., 2019; Al-Hamashi et al., 2020), which determine substrate specificity and the type of methylated products. Tau methylation has been identified at multiple residues, with at least eleven sites reported under physiological conditions, many located within the MTBR (Funk et al., 2014; Balmik and Chinnathambi, 2021). Methylation appears to decrease as tau aggregates, with only seven sites detected in PHF tau (Funk et al., 2014). Functional studies suggest that mono- and di-methylation can reduce tau nucleation, elongation, and aggregation propensity (Funk et al., 2014). Methylation may also compete with other lysine-directed PTMs such as ubiquitination, acetylation, and SUMOylation (Balmik and Chinnathambi, 2021). For example, methylation at K254 can inhibit ubiquitination at the same site and potentially affect tau degradation (Kontaxi et al., 2017; Thomas et al., 2012), while methylation at nearby KXGS motifs, such as K267, may affect nearby phosphorylation events, including S262 phosphorylation (Thomas et al., 2012).

### Lactylation

Protein lactylation is an emerging lysine modification that links cellular metabolism to protein regulation. In this process, a lactyl group derived from lactyl-CoA is added to lysine *ε*-amino groups, a reaction catalyzed by p300/CBP and removed by HDAC1/3 or sirtuins (Han et al., 2025). Since lactylation introduces a bulky and negatively charged group, it can alter protein charge distribution and potentially affect protein interactions and structural properties.

Recent studies suggest that lactylation may contribute to tau pathology in AD. Quantitative proteomic analyses identified increased tau lactylation in AD brain tissue, particularly at residue K331, with modification levels correlating with Braak stage progression (Zhang et al., 2025). Elevated cerebrospinal fluid lactate levels have also been reported in early AD, suggesting metabolic dysregulation associated with disease progression (Cao et al., 2025; Liguori et al., 2015). In addition, lysine lactylation has been implicated in ferroptosis pathways, where it may disrupt interactions between tau and iron transporters, leading to iron accumulation and neuronal toxicity (An et al., 2024). However, the mechanistic consequences of tau lactylation for tau aggregation, clearance, and neuronal dysfunction remain largely unexplored.

### UFMylation

UFMylation is a ubiquitin-like PTM that contributes to cellular homeostasis and has emerging roles in neurodevelopmental and stress responses (Komatsu et al., 2024; Millrine et al., 2023; Gerakis et al., 2019; Nahorski et al., 2018; Ni et al., 2021). Similar to ubiquitination, UFMylation occurs through an enzymatic cascade involving the E1 activating enzyme UBA5, the E2 conjugating enzyme UFC1, and the E3 ligase UFL1, which together conjugate the ubiquitin-fold modifier UFM1 to lysine residues on target proteins (Komatsu et al., 2004; Tatsumi et al., 2010). This pathway regulates multiple processes relevant to neurodegeneration, including DNA damage responses, ER stress, autophagy, and immune signaling (Ciccia and Elledge, 2010; Yoo et al., 2014; DeJesus et al., 2016; Chung and Yoo, 2022). Consistent with these roles, defects in DNA repair pathways have been observed in human AD brains and animal models, potentially contributing to tau hyperphosphorylation and neuronal apoptosis (Shackelford, 2006; Kanungo, 2016; Welch and Tsai, 2022; Asada-Utsugi et al., 2022). Recent work has also linked UFMylation directly to tau biology. Genes in the UFMylation pathway, including UFM1, UFL1, and DDRGK1, are required for physiological tau oligomer formation, and subsequent studies suggest that UFMylation may positively regulate tau propagation (Samelson et al., 2024; Parra Bravo et al., 2024a). Recent analyses of human AD brain tissue further revealed increased levels of conjugated UFM1, indicating hyper-UFMylation that correlates strongly with pathological tau accumulation in affected brain regions. These findings suggest that dysregulation of the UFMylation pathway may act as a modifier of tau pathology and may intersect with neuronal stress pathways, including the DNA damage response and unfolded protein response (Yan et al., 2024). However, the mechanisms through which UFMylation influences tau structure, aggregation, or intercellular spread remain poorly understood.

### O-GlcNAcylation

O-GlcNAcylation is a reversible PTM in which an N-acetyl-glucosamine moiety is attached to serine or threonine residues of intracellular proteins (Spiro, 2002). This process is a specialized, non-canonical form of glycosylation that is regulated by O-GlcNAc transferase, which adds the sugar group, and O-GlcNAcase, which removes it (Yang and Qian, 2017). Since O-GlcNAcylation occurs on serine and threonine residues that are also targets for phosphorylation, these two PTMs often interact competitively or reciprocally. Several O-GlcNAc sites have been identified on tau, including T123, S208, S238, S400, and one of S409, S412, or S413 (Arnold et al., 1996; Gong et al., 2016; Yuzwa et al., 2012; Yuzwa et al., 2011; Smet-Nocca et al., 2011). In the AD brain, tau O-GlcNAcylation is reduced, whereas tau phosphorylation is increased (Liu et al., 2004; Robertson et al., 2004). Experimental studies indicate that increasing O-GlcNAc modification can enhance tau microtubule binding, promote tau degradation, and suppress tau aggregation (Arnold et al., 1996; Yuzwa et al., 2014). Nevertheless, important questions remain regarding how O-GlcNAcylation contributes to tau regulation during normal aging and how it interacts with phosphorylation and other PTM networks in neurodegenerative disease.

## Interplay among tau PTMs

**Figure 3:**
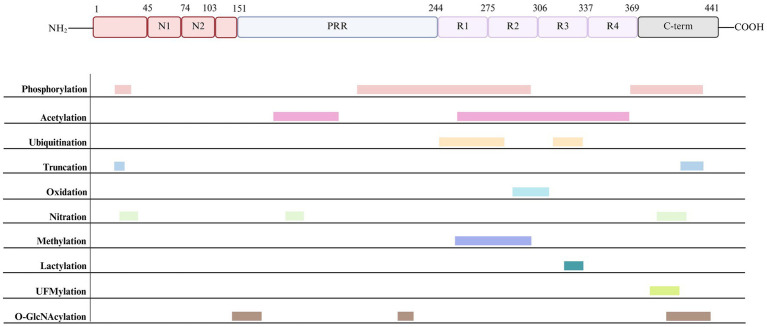
Distribution of tau PTMs across the full-length protein. Top panel illustrates the domain organization of full-length tau, including the N-terminal projection domain (N1 and N2), proline rich region (PRR), microtubule binding repeats (R1 to R4), and C-terminal domain. Bottom panel shows the approximate locations of residues along the tau sequence reported to undergo the PTMs discussed in this review. Created with BioRender.com.

PTMs rarely operate in isolation. Instead, tau function is shaped by extensive crosstalk among multiple PTMs that collectively regulate its structure, interactions, and turnover. This combinatorial regulation is constrained by the special organization of modification sites along the tau sequence. As the number of modification sites increases, the complexity of these regulatory relationships expands substantially. Large-scale proteomic analysis highlights the remarkable density of tau modifications. For example, analysis of human tau isolated from brain tissue across stages of AD identified 95 distinct PTMs affecting 88 residues, illustrating the extraordinary combinatorial landscape through which tau regulation occurs (Wesseling et al., 2020). Importantly, many PTMs occur on overlapping or neighboring residues, creating opportunities for cooperative or antagonistic interactions that can either stabilize physiological tau function or promote pathogenic transitions (Figure 3).

One of the most extensively studied PTM relationships involves phosphorylation and acetylation, which frequently cooperate to promote tau dysfunction. Cohen et al. (2013) demonstrated that tau possesses intrinsic acetyltransferase activity and is capable of autoacetylation. Mimicking phosphorylation at KXGS motifs, including S262 and S356, enhanced this acetyltransferase activity, suggesting that phosphorylation can promote downstream acetylation events (Cohen et al., 2013). Consistent with this idea, acetylation at K280 within the MTBR is strongly associated with insoluble tau aggregates derived from diseased tissue (Cohen et al., 2011). Blocking K280 acetylation reduces downstream acetylation at K369 as well as phosphorylation at S262 and S356, suggesting that acetylation at this site may act as a priming event for additional pathogenic modification (Tseng et al., 2021). However, PTM interactions are not universally synergistic. For example, acetylation at K321 has been shown to block phosphorylation at S324, indicating that certain acetylation events may counteract phosphorylation-dependent toxicity (Carlomagno et al., 2017). Additional mechanistic work from the same study further identified an HDAC6-regulated acetylation-phosphorylation switch, in which acetylation-mimic substitution at K321 (K321Q) reduced tau filament assembly and prevented downstream phosphorylation at S324, whereas K274Q produced a smaller but significant reduction in microtubule assembly (Carlomagno et al., 2017). Complementary evidence indicates that acetylation can more broadly reshape phosphorylation networks *in vivo*. In a human tau knock-in Drosophila model expressing physiological tau levels, acetylation-mimicking at multiple residues, including K163, K280, K281, and K369, produced widespread reductions in phosphorylation while selectively increasing phosphorylation at S262 and weakening microtubule binding (Gorsky et al., 2017).

Another well-characterized interaction occurs between phosphorylation and O-GlcNAcylation, which frequently exhibits reciprocal regulation. Early studies examining diabetes and AD suggested that reduced O-GlcNAcylation exposes tau to increased kinase activity, promoting hyperphosphorylation, neurofibrillary tangle formation, and neuronal dysfunction (Dias and Hart, 2007). Subsequent work confirmed this inverse relationship between the two modifications (Deng et al., 2008). However, site-specific analyses indicate that this interaction is more complex than simple competition. For example, oxidative stress-induced increases in intracellular O-GlcNAc levels were associated with reduced phosphorylation at S199 and PHF-1 epitopes (S396/S404), while phosphorylation at S262 increased (Katai et al., 2016). The findings suggest that the relationship between phosphorylation and O-GlcNAcylation may depend on local structural context and cellular stress conditions.

Interactions between phosphorylation and nitration also illustrate how redox-linked PTMs can influence tau modification networks. Several tyrosine residues on tau, including Y18, Y29, Y197, and Y394, can undergo nitration, with Y18, Y29, and Y394 commonly detected in disease-associated contexts (Reyes et al., 2011; Reynolds et al., 2006). Since these residues are also targets for phosphorylation, potential competition between the two modifications has been proposed. However, experimental evidence suggests that nitration and phosphorylation may instead act synergistically in some contexts. Peroxynitrite exposure induces both nitration and tau oligomerization while impairing microtubule binding activity (Zhang et al., 2005). Similar treatments in animal models promote tau hyperphosphorylation, nitration, and aggregation simultaneously (Zhang et al., 2006). Additional work indicates that phosphorylation at Y29 mediated by TYK2 enhances tau aggregation and facilitates nitration at the same residue, suggesting that structural changes introduced by one modification can promote additional PTMs that further destabilize tau (Kim et al., 2024).

Lysine residues represent particularly important nodes for PTM interplay because they can be modified by multiple competing modifications, including acetylation, ubiquitination, and methylation. Residues such as K163, K174, and K180 have been reported as acetylated in disease contexts but methylated under conditions associated with reduced aggregation propensity (Min et al., 2010; Huseby et al., 2019). Since these modifications are mutually exclusive at the same residue, they create competitive regulatory interactions that influence tau stability and aggregation behavior. Consistent with this idea, methylated tau species are reduced in AD brain tissue, and this reduction correlates with tau aggregation and NFT formation (Balmik and Chinnathambi, 2021). In some cases, methylation may directly interfere with other PTMs. For example, methylation at K254 has been proposed to block ubiquitination at the same residue, potentially altering tau degradation through the ubiquitin–proteasome system (Thomas et al., 2012). Similarly, methylation at K290 has been detected in healthy control brains, whereas ubiquitination at the same site is more frequently associated with disease states (Morris et al., 2015).

Interactions between acetylation and ubiquitination further highlight the complexity of tau proteostasis regulation. Early studies demonstrated that deletion of the deacetylase SIRT1 increased tau acetylation and phosphorylation while reducing tau polyubiquitination and clearance (Min et al., 2010). Conversely, SIRT1-mediated deacetylation promoted tau ubiquitination and proteasomal degradation (Min et al., 2010). Subsequent work has suggested that pharmacological modulation of deacetylases can influence these pathways in different ways. For example, HDAC6 inhibitors have been shown to alter tau acetylation and promote ubiquitination-dependent degradation of pathological tau species (Fan et al., 2018; Choi et al., 2020). One proposed mechanism involves acetylation-dependent recruitment of molecular chaperones and ubiquitin ligases that facilitate proteasomal targeting (Choi et al., 2020). However, differences in the biological roles of distinct deacetylase families may explain why some studies report opposing outcomes when acetylation pathways are manipulated (Park and Kim, 2020).

Collectively, these observations illustrate that tau PTMs form a highly interconnected regulatory network rather than a series of independent chemical events. Individual modifications can act as priming signals, competitive inhibitors, or cooperative amplifiers that reshape tau conformation, stability, and aggregation propensity. Understanding how these modifications interact across different residues and cellular contexts remains a major challenge, and future work will be required to determine how PTM networks collectively drive the transition of tau from a functional neuronal protein to a pathogenic aggregate-forming species.

## Conclusion

Tau biology is increasingly understood through the lens of post-translational modification networks that dynamically regulate its structural and functional properties (Wang and Mandelkow, 2016; Alquezar et al., 2020). In healthy neurons, tau operates as a highly adaptable intrinsically disordered protein whose interactions with microtubules, membranes, nucleic acids, and signaling complexes are tuned by a diverse array of PTMs. Rather than functioning as isolated binary switches, these modifications collectively reshape tau conformation, interaction landscapes, subcellular localization, and turnover. In this way, PTMs provide a flexible regulatory system that allows tau to transition between functional properties required for neuronal homeostasis.

In neurodegenerative diseases, however, this finely balanced PTM landscape becomes destabilized. Aberrant phosphorylation, acetylation, ubiquitination, truncation, and redox-linked modifications accumulate and interact in ways that promote tau mislocalization, impaired clearance, and conversion into aggregation-prone conformations. Increasing evidence suggests that tau pathology does not arise from a single dominant modification, but rather from coordinated shifts in PTM networks that collectively bias tau toward pathogenic configuration (Wesseling et al., 2020). Crosstalk among PTMs can determine whether tau remains soluble or forms oligomers and fibrils, whether it is efficiently degraded or persistently accumulates, and whether it remains functionally integrated within neuronal systems or acquires prion-like propagation properties (Iqbal et al., 2010; Wegmann et al., 2021).

Despite substantial progress in mapping tau PTMs, major knowledge gaps remain. Importantly, most studies have focused on individual PTMs in isolation, whereas tau exists in a highly modified and combinatorial form *in vivo* (Wesseling et al., 2020; Morris et al., 2015). Many modification sites have been cataloged but lack mechanistic interpretation, and the temporal order and combinatorial logic by which multiple PTMs cooperate to reshape tau function remain poorly understood. Addressing these questions will require integrative approaches that combine high-resolution proteomics, structural biology, cellular models, and *in vivo* systems capable of resolving PTM dynamics across disease progression. Emerging technologies, including quantitative PTM profiling, chemical biology tools to manipulate specific modifications, and cryo-EM analysis of disease-associated tau assemblies (Arakhamia et al., 2020; Kumar et al., 2026), will be instrumental in defining how PTM networks regulate tau transitions. In addition, emerging computational and data-driven approaches, including machine learning and AI-based modeling, may enable integration of complex PTM datasets to identify combinatorial modification patterns, predict functional outcomes, and guide precision therapeutic strategies. Importantly, additional PTMs not extensively discussed here, including citrullination, glycation, succinylation, and SUMOylation, may further expand the complexity of tau regulation and warrant deeper investigation (Liang et al., 2025; Bondar et al., 2025; Chai et al., 2025; Chinnathambi and Rangappa, 2025).

Understanding tau PTMs as components of a regulatory code has important therapeutic implications. Targeting individual modifying enzymes, such as kinases, acetyltransferases, deacetylases, and proteases, with small molecule therapeutics has demonstrated promise in preclinical models, but yielded limited clinical benefit in subsequent trials, consistent with the complexity of tau PTM regulation. For example, Tideglusib, a GSK3β kinase inhibitor, was used to treat patients with mild to moderate AD for 26 weeks but ultimately provided no clinical benefits (Lovestone et al., 2015). Another clinical trial investigated a Fyn kinase inhibitor, named saracatinib, which was safe and well tolerated in a Phase Ib trial (Nygaard et al., 2015), but did not benefit disease pathology during Phase IIa trials (van Dyck et al., 2019). Similarly, Ceperognastat, an O-GlcNAcase inhibitor, showed promising preclinical results (Yuzwa et al., 2012; Graham et al., 2014; Hastings et al., 2017), and underwent four Phase I trials before moving onto Phase II. However, participants receiving the treatment exhibited faster cognitive decline compared to the placebo group and experienced adverse effects resembling drug-induced neurotoxicity compared to the placebo group, which resulted in the trial ending (Meade et al., 2026; Kielbasa et al., 2024).

These trials primarily target individual tau PTM enzymes. However, given that tau pathology arises from coordinated shifts across PTM networks rather than a single modification, it is not surprising that such approaches have shown limited clinical efficacy. Future strategies may need to move beyond single-enzyme interventions and adopt combinatorial approaches that restore a broader balance of PTMs and proteostasis. A complementary approach is the direct targeting of tau species defined by disease-associated PTM signatures. Monoclonal antibodies that recognize specific modified forms of tau, including acetylated tau, may enable selective neutralization of pathogenic conformations while sparing physiologically modified tau (Martín-Ávila et al., 2025; BryanIii et al., 2024; Chai et al., 2011). Such strategies could promote clearance of intracellular pathological tau or scavenge extracellular tau species that drive intercellular propagation. Defining how aging, metabolic stress, and proteostasis decline reshape the tau PTM landscape will therefore be critical for understanding neuronal vulnerability to tau pathology and for developing therapies that selectively target the most pathogenic tau species.

Together, the emerging view is that tau PTMs operate as an integrated regulatory system that governs tau function across physiological and pathological contexts. Deciphering this modification network will not only deepen our understanding of tau biology but also provide new opportunities to intervene in the molecular processes that drive tau-mediated neurodegeneration.
